# Decision Making in Severe Equine Asthma—Diagnosis and Monitoring

**DOI:** 10.3390/ani13243872

**Published:** 2023-12-16

**Authors:** Joana Simões, Paula Tilley

**Affiliations:** 1 Equine Health and Welfare Academic Division, Faculty of Veterinary Medicine, Lusófona University, Campo Grande 376, 1749-024 Lisbon, Portugal; 2 CIISA-Centre for Interdisciplinary Research in Animal Health, Faculty of Veterinary Medicine, University of Lisbon, 1300-477 Lisbon, Portugal; ptilley@fmv.ulisboa.pt; 3 Associate Laboratory for Animal and Veterinary Sciences (AL4Animals), Faculty of Veterinary Medicine, University of Lisbon, 1300-477 Lisbon, Portugal

**Keywords:** equine, severe equine asthma, diagnosis, clinical history, clinical signs, diagnostic imaging, lung function, cytology, decision making

## Abstract

**Simple Summary:**

Severe equine asthma (SEA) is an important respiratory disease affecting a large number of horses worldwide. This chronic disease induces cough and respiratory distress compromising the animals’ athletic ability and welfare. A wide variety of diagnostic tests have been described for researching and diagnosing SEA, but not all are commonly available outside of large research or specialized diagnostic centers. Moreover, in routine ambulatory practice the process of decision making is not always easy, especially in more complex medical cases, but its importance is pivotal for individualized patient care. Thus, the aim of this paper is to develop a flow-chart to assist equine practitioners in the process of decision making associated with diagnosing and monitoring SEA.

**Abstract:**

Decision making consists of gathering quality data in order to correctly assess a situation and determine the best course of action. This process is a fundamental part of medicine and is what enables practitioners to accurately diagnose diseases and select appropriate treatment protocols. Despite severe equine asthma (SEA) being a highly prevalent lower respiratory disease amongst equids, clinicians still struggle with the optimization of routine diagnostic procedures. The use of several ancillary diagnostic tests has been reported for disease identification and monitoring, but many are only suitable for research purposes or lack practicality for everyday use. The aim of this paper is to assist the equine veterinarian in the process of decision making associated with managing SEA-affected patients. This review will focus on disease diagnosis and monitoring, while also presenting a flow-chart which includes the basic data that the clinician must obtain in order to accurately identify severely asthmatic horses in their everyday routine practice. It is important to note that European and American board-certified specialists on equine internal medicine can provide assistance in the diagnosis and treatment plan of SEA-affected horses.

## 1. Introduction

Severe equine asthma (SEA) is a chronic insidious respiratory disease which commonly affects mature adult horses [1,2]. Its estimated prevalence in the northern hemisphere is 20%, but the number of affected individuals continues to rise [3,4,5].

The precise immunological pathways of this multifactorial disease are complex and not yet fully understood [6,7,8,9,10,11,12], but it is known that when susceptible individuals are exposed to high concentrations of respirable particles they develop inflammation, bronchospasm and airway hyperreactivity [13,14,15,16]. Consequently these animals develop increased respiratory effort at rest, cough and nasal discharge which, depending on inflammation severity, may impact athletic performance and the horse’s well-being [17,18].

According to the type of inflammatory triggers associated with SEA exacerbation, two major disease phenotypes have been described [1]. The stable-associated SEA mainly involves exposure to organic respirable particles found indoors during the colder months, such as in bedding materials, hay and straw [19,20,21], whilst the pasture-associated SEA occurs in animals kept at pasture during the warmer season due to exposure to pollen [22,23,24].

However, inflammation can be triggered by a large number of molecules with a synergistic effect, such as LPS, pollen, mites, fungi spores, or even plastic particles, which can be found in the horses’ habitat [19,20,21,25,26,27,28]. Because antigen avoidance can be extremely difficult to achieve, affected animals tend to present recurrent episodes of disease exacerbation [29].

Disease diagnosis is not always linear, and many practitioners struggle with decision making when confronted with a potential severely asthmatic horse. Empirical treatment, lack of compliance and erroneous posology are just some of the culprits for an unsuccessful treatment response [29], but misdiagnosis can further hinder this process [30]. Thus, thorough knowledge of the available diagnostic and therapeutical management options for SEA can help improve disease outcome. This paper provides a review of the currently available diagnostic tools, whilst offering a flow-chart for decision making in routine equine veterinary practice.

## 2. Diagnosis

In evidence-based medicine, reaching a correct diagnosis is paramount to produce individualized patient care and optimize clinical outcome [31]. Every patient is unique, as their medical conditions can vary in severity, underlying causes, and treatment response. Thus, by accurately identifying the patient’s condition, veterinarians can tailor their treatment plans to address the specific needs of the animal and their owner, taking into account risks and potential benefits, ultimately maximizing the chances of therapeutic success and animal well-being [29,32]. Therefore, diagnosis must be sought using the most up-to-date research and clinical guidelines in the decision-making process. This approach ensures that patients are subjected to the most relevant clinical interventions and receive treatments that have been proven effective [30,31]. Also, regarding the practitioner, evidence-based medicine is also the best way to efficiently allocate resources and contribute to continuous learning and improvement in healthcare practice.

### 2.1. History, Clinical Signs and Clinical Scores

Severely asthmatic horses will have a history of recurrent respiratory disease with clinical signs being triggered by exposure to specific environmental factors [1]. Often these signs will be seasonal with coughing or labored breathing occurring during spring or autumn. Some horses will also develop clinical signs when performing physical exercise in dusty arenas or when moved to stables with poor air quality [33,34] which is frequently associated with dusty bedding materials, hay or straw and poor ventilation [35,36,37]. Thus, it is important to carefully examine the horses’ environment but also to inquire about any recently made changes, since some owners may have decided to alter the animals’ environment but have forgotten to report it during the initial consultation. Most commonly, owners will report that in response to being fed poor quality hay the horse will exhibit bouts of cough [38,39]. Two different phenotypes have been reported in SEA which means that asthmatic horses can develop clinical signs when exposed to stable environmental conditions (fungal spores, organic dust, mites, endotoxins, among others) or when exposed to a pasture environment (pollen and fungal spores) [4,19,40,41]. Identifying the horses’ phenotype will determine the best treatment protocol for these animals, and intradermal testing can be useful for constructing an individualized allergen eviction protocol [42,43].

The clinical signs associated with SEA are quite distinctive [39,44], which can lead to the temptation of making a presumptive diagnosis based only on the patient’s clinical history and physical examination. Nonetheless, this should be discouraged since it can lead to an erroneous diagnosis especially in horses examined during disease remission or with low grade airway hyperreactivity.

Severely asthmatic horses have a history of chronic and persistent cough, which can be seasonal, associated with stabling and hay feeding or pasture [45]. Close inspection of the affected horses’ living quarters and feed will normally reveal the triggering factor.

Airway inflammation, hyperreactivity and obstruction result in cough, exercise intolerance, increased respiratory effort at rest and nasal discharge [17,43,46], and the severity of the exhibited clinical signs tend to correlate with the degree and persistence of the inflammatory process [18,47,48].

Cough is usually the first clinical sign reported by owners and although the onset of exercise intolerance precedes it [49], the latter is not always reported early on. This could be related to the expected athletic level of the affected horse, since low grade inflammation may not impair the performance of less strenuous physical activities [50]. With disease progression the severity of cough increases and paroxysmal bouts of cough are typically observed [39,51]. 

The associated airway obstruction results in overt respiratory distress with consequent modification of the respiratory pattern, characterized by a short inspiration followed by prolonged exhalation and abdominal lift [52]. Affected animals present increased abdominal effort, with consequent hypertrophy of the external oblique muscles (‘heave line’) and nasal flaring as an attempt to reduce upper airway resistance [17,18,47,53].

Changes in mucus rheology along with its decreased clearance result in the accumulation of secretions in the tracheobronchial tree [54,55]. However, due to the regular swallowing of these secretions, horses only exhibit nasal discharge occasionally [56]. Nonetheless the presence of a tracheal rattle can be easily auscultated by placing the stethoscope over the tracheal area during physical examination, indicating the presence of mucus [57]. Since these animals are also at risk of contracting opportunistic respiratory infections, some horses may develop a purulent nasal discharge [58,59]. 

Thoracic auscultation of affected animals will reveal increased bronchovesicular sounds, end-expiratory wheezes due to airway narrowing, and inspiratory crackles caused by the opening of collapsed airways. An expanded pulmonary field can also be auscultated due to lung hyperinflation, secondary to air entrapment in the alveoli [1,2,60]. In some cases, lung auscultation will be unusually silent despite the horse exhibiting substantial respiratory effort due to a remarkable compromise of airflow associated with significant airway obstruction [60].

Loss of body mass and cachexia can also occur in horses with extreme respiratory distress due to a combination of reduced food intake and increased energy expenditure to overcome the expiratory obstruction [50,61,62].

Clinical scores can help assess and monitor disease severity and prognosis by evaluating common clinical signs observed during disease exacerbation, such as cough, nasal flaring, nasal discharge, abdominal lift, and exercise intolerance [17,18,46,63]. 

However, these scores alone can be insufficient, since mild or subclinical cases of severe equine asthma have little or no clinical signs despite maintaining some degree of airway inflammation and hyperreactivity [39,44,45,64]. Although scores based on nasal flaring and abdominal effort correlate with airway dysfunction, exercise intolerance does not, and cough may not occur during disease remission [47,53,65]. Examples of these scores are the ones published by Rush [65,66], Robinson [53,67] and Tesarowski [68,69].

The main limitation of clinical sign scores is therefore the correct diagnosis of severely asthmatic horses when in disease remission, making other ancillary diagnostic tests crucial for accurate disease characterization and exclusion of other alternative diagnoses [44,46,53]. Nonetheless, they can be useful in the initial triage and for the continuous assessment of treatment response, without resorting to more invasive diagnostic methods.

Furthermore, methods like the Horse Owner Assessed Respiratory Signs Index (HOARSI) [39,46], and the Visual Analog Scale (VAS) [70], can be particularly useful for triage since they rely on information reported by horse owners to help identify the presence of respiratory disease. The HOARSI questionnaire scoring system is based on clinical history and on cough frequency, nasal discharge, breathing at rest and the horse’s performance [15]. It has been primarily used to identify diseased asthmatic horses (with mild, moderate or severe equine asthma) and has shown good validation and repeatability [14,39,46]. However, it does not provide information on the degree of severity of each clinical entity and may misdiagnose horses in disease remission or in early inflammatory stages of SEA.

### 2.2. Diagnostic Imaging

Radiography and ultrasonography are routinely used ancillary diagnostic tests in equine ambulatory practice, since both are non-invasive and well accepted by horses [71,72].

In SEA, the radiographic findings have been found to correlate with the clinical signs and disease severity [17]. SEA-affected horses exhibit lung pattern changes, such as increased bronchovascular, interstitial and bronchial interstitial lung patterns, along with thickening of tracheal and bronchial walls [17,73]. In extreme cases, when disease progression results in lung remodeling, interstitial infiltration, increased lung radiopacity and bronchiectasis can also be observed [74,75]. Unfortunately thoracic x-rays are not able to detect discrete lung inflammation [18,76]; their interpretation can be challenging due to the superimposition of several anatomic structures and to fully observe the equine lung, x-rays of at least four different thoracic fields are required [77,78].

Ultrasound examination allows the clinician to attain more detailed information about the surface of the lung, including the assessment of effusion and its characteristics. However the most common ultrasonographic finding in severely asthmatic horses is the presence of comet tails which are non-specific artifacts associated with inflammation [79,80,81]. Alternatively, endobronchial ultrasound (ultrasonography via endoscopy) can be used to determine whether bronchial remodeling, a common feature of SEA, is present [82,83]. Using this ancillary test, one can distinguish SEA-affected horses in exacerbation from healthy individuals, but it fails to correctly identify severely asthmatic horses in remission [82].

Because the radiographic and ultrasonographic changes observed in severely asthmatic horses are not pathognomonic of the disease [76,80,81], it has been proposed that imaging is not required for the diagnosis of SEA in an ambulatory setting [2]. Nonetheless, the authors believe that these two techniques should be considered for disease staging and for excluding other potential differential diagnoses [1,17,71,72].

### 2.3. Endoscopy

Respiratory endoscopy alone or when combined with other sampling techniques can provide insight about the equine upper and lower airways [54,84,85]. Thus it can contribute to the characterization of SEA and to the exclusion of other differentials [1,17,55,85].

Endoscopic examination of severely asthmatic horses will reveal the presence of mucus in the trachea and bronchi and thickening of the carina (tracheal septum). The presence of bronchospasm and mucosal hyperemia associated with inflammation can also be assessed [17,55,86,87,88,89,90].

Tracheal secretions will usually form a small pool at the lowest point of the trachea. However, decreased mucus clearance and altered rheology increases the amount of mucus observed and mucus strings can be observed in the lateral and dorsal aspects of the trachea [55,91].

Most endoscopic scoring systems assess the quantity and quality of tracheal mucus [17,86,87,88,90], and mucus accumulation was found to correlate with clinical scores [92], cough frequency [51] and cytological indicators of airway inflammation [55]. The cytological findings associated with SEA are described in Section 2.5.

During disease remission, the amount of secretions found in the trachea of asthmatic horses significantly decrease and endoscopic mucus scores alone do not differentiate these animals from healthy ones [18,46]. Furthermore, mucus accumulation is not a sensitive indicator of SEA since it is also present in other diseases, including mild and moderate equine asthma [1,84].

### 2.4. Lung Function Tests

Pulmonary function testing has become an important tool in the evaluation of the respiratory system since it provides information about ventilation and the dynamics of this system [71,93]. They are considered the gold standard for the diagnosis of asthma both in human and equine medicine [94]. In SEA they contribute to disease diagnosis and assessment of disease severity as well as treatment response. Nevertheless, no single test can be considered perfect, as they all have their strengths and weaknesses, and their selection will be determined by the clinician’s needs, their practicality and availability. Moreover, despite technological evolution, some testing equipment are not suitable options for ambulatory settings, which is why lung function testing is mostly available at large research centers with few commercial options available in the market [2].

Arterial blood gas analysis assesses lung gas exchange and can be used in an ambulatory setting [95]. Severely asthmatic horses commonly present only hypoxemia, but lower values of pH and increased values of PaCO_2_ have also been reported [18,96,97,98,99]. Its main disadvantage is that it lacks sensitivity for recognizing animals in remission [99].

The change in pleural pressure (ΔPpl), assessed by an oesophageal balloon catheter, is considered the gold standard for the diagnosis of SEA. The measured values can be interpreted on their own, with ΔPpl > 15 cm H_2_O being considered the cut off value for disease diagnosis [1,46]. This method can easily be used in an ambulatory setting, but in the authors’ experience some owners may be reticent to allow this test to be performed on their horses due to its invasive nature. When combined with a pneumotachograph, standard lung mechanics can be assessed, namely dynamic compliance (Cdyn), pulmonary resistance (RL), and work of breathing (W). Airway obstruction results in increased RL, W and ΔPpl, and decreased Cdyn [1,53,100]. Unfortunately ΔPpl alone will not help differentiate healthy animals from severely asthmatic ones in remission, nor will standard lung mechanics, as it has been reported to have a similar sensitivity to a clinical exam [44,53,63,101]. However, when combined with histamine bronchoprovocation, its sensitivity improves and, despite its limitations, it remains a valuable tool for assessing respiratory function in an ambulatory setting [102].

Flowmetrics is based in boxless plethysmography and it combines respiratory inductance plethysmography (RIP) with pneumotachography [103]. This system, which has been specifically developed for equines and was suited for field testing [104], is no longer commercially available. It had a sensitivity similar to that of pleural pressure but, when combined with histamine bronchoprovocation, allowed the detection of horses in disease remission [18,46,105,106].

Lung function testing can be associated with either a histamine or bronchodilator challenge [93]. Airway hyperreactivity, the reversible narrowing of airways in response to a bronchoconstrictor stimulus, such as histamine, occurs in all asthmatic horses, especially during disease exacerbation but is also present in cases of mild obstruction without apparent clinical signs [1,18,46,104,105,107,108,109]. After a base-line reading, increasing concentrations of a histamine solution (0, 2, 4, 8, 16 and 32 mg/mL) are progressively nebulized and following each administration, lung function is re-evaluated. If significant bronchoconstriction is observed or if clinical discomfort is noted, the test is immediately stopped. A healthy horse will tolerate the inhalation of the maximum histamine concentration, whilst asthmatic horses will tolerate significantly lower doses [18,46].

When asthmatic horses present with a severe compromise of baseline pulmonary function, it is recommended to perform a bronchodilator challenge instead [2,62]. A bronchodilator is administered (e.g., albuterol 450–900 μg) after a base-line reading and 15 min later pulmonary function is re-assessed [1,93]. Within 10 min, a 50% improvement of airway resistance should occur in SEA-affected horses in exacerbation [93]. Furthermore, both the maximum value and the magnitude of the observed bronchodilation can also predict, to some degree, the future therapeutic response.

The use of spirometry [44,110,111,112], electrical impedance tomography [113], and impulse oscillation system (IOS) for assessing the dynamics of equine lower airways have shown promising results, particularly IOS, which has been reported to differentiate severely asthmatic horses in remission from healthy controls [114,115,116,117]. At the present time these tests are more suited for research purposes and have yet to be perfected for everyday clinical use. Nonetheless, the authors believe that, similar to human medicine, lung function testing will become an indispensable diagnostic tool to monitor lower airway inflammation and assist in the diagnosis and staging of SEA.

### 2.5. Cytology

In equine ambulatory medicine, airway cytology remains a fundamental technique for diagnosing and monitoring SEA. It provides insight into the inflammatory status of airways and although it is not considered the gold standard for the diagnosis of this disease, its practicality in an ambulatory context has rendered this ancillary diagnostic test popular. Cytological samples can be collected using a wide variety of methods, including brush cytology, tracheal wash (TW), bronchoalveolar lavage fluid (BALF) or even bronchial biopsies [118,119,120,121]. 

Of these methods, BALF cytology is considered to be the one which most accurately reflects the cellular populations of the bronchi and alveoli and the consequent degree of inflammation found in the horses’ lungs [120,122,123]. Sampling can easily be performed transendoscopicaly or ‘blindly’, via a balloon catheter, by instilling a volume of 250 to 500 mL of saline and a minimum of 400 cells should be counted [2,18,120,124,125]. When endoscopically aided, the BALF collection can be guided in order to sample either the right or left lung. This option can be useful if a localized disease or lesion is suspected (i.e., a lung abcess), but is unnecessary in SEA-affected horses where both lungs are equally affected [124].

BALF samples of healthy animals have <400 cells/μL and a superficial foam layer, indicating the presence of pulmonary surfactant. Alveolar macrophages (40–70%) and lymphocytes (30–60%) are the most commonly observed immune cells followed by neutrophils (<5%), mast cells (<2%) and eosinophils (<1%) [1]. The cytological profile of the severely asthmatic horse is usually characterized by neutrophilia (>20% neutrophils) and a reduction in macrophage and lymphocyte percentages [1,17,18,126,127,128]. An increased amount of mucus is also observed which can form Curschmann’s spirals [57,129].

BALF differential cell counts correlate well with airway obstruction and hyperresponsiveness, and a higher percentage of neutrophils is associated with greater disease severity, coughing and worse mucus scores [17,47,51,129,130]. During clinical remission, affected horses maintain a slightly elevated neutrophil percentage [63], and despite corticosteroid treatment, BALF neutrophilia can persist when severely asthmatic horses continue to be exposed to the offending respirable particles [40,131,132,133].

Furthermore, it has been reported that the percentage of neutrophils in BALF can be used to classify the severity of SEA, as it correlates well with the clinical signs exhibited by asthmatic horses, changes observed in thoracic x-rays, mucus scores determined through endoscopy and airway remodeling [17,127]. It is important to mention that the bronchoalveolar lavage procedure should always be conducted after assessing lung function, as it has been shown to temporarily improve pulmonary resistance (RL), likely due to mucus clearance [134].

However, BALF cytology is not well accepted for the evaluation of high performance athletes [135]. Despite the reported poor correlation between neutrophil counts in the TW and in the BALF, which may indicate that the cellular population found in the tracheal lumen is not representative of lower airways [123,136,137], British race horse veterinarians prefer to perform TW instead of BALF collection [2,135]. Conversely, a study on a population of 154 horses showed a good correlation between the neutrophil percentage in TW and BALF cytology with only 17.5% of the cases having been classified differently [138]. Nonetheless, until more studies are performed, BALF remains more suitable for the accurate diagnosis of SEA and when feasible, the combination of both TW and BALF can aid in disease diagnosis and characterization.

Diagnostic cytology should not be performed if the horse is receiving corticosteroids, since this will invariably modulate the inflammatory response associated with the disease and can result in an incorrect diagnosis. Furthermore, it should be noted that neutrophilia is not a prerequisite for the lung function deterioration observed in SEA and some horses may present an increased respiratory effort without BALF neutrophilia. Therefore, BALF cytology should not be used alone for diagnosing SEA [83,126,139].

The clinician should also be aware that signs of neutrophilic degeneration associated with the presence of bacteria or fungal spores/hyphae, either free or phagocyted, can be an indicator of a primary infectious condition or of an opportunistic infection. In such cases bacterial or mycological cultures should be considered to identify the causal agent and adjust the therapeutic protocol. 

### 2.6. SEA Staging

One of the main challenges of managing a severely asthmatic patient is continuously monitoring disease severity and response to treatment. An initial detailed characterization of the disease may help select the best therapeutic protocol and optimize environmental management catering to the horses’ and owners’ individualized needs [94].

Staging methods were developed to help clinicians gather information about disease severity by combining history, physical examination and a variety of ancillary diagnostic tests. 

A relatively complete SEA clinical staging method has been published, which encompasses clinical history reported by the owners and clinical signs observed during clinical examination, namely cough frequency, nasal flare and abdominal lift. It also uses ancillary diagnostic tests to quantify airway inflammation and remodeling, such as thoracic x-ray, endoscopy and BALF cytology [17]. This staging method has to be carried out in a hospital and only evaluates the present condition of the horse and does not take into account reported history. It also does not evaluate lung function which has been considered an important diagnostic indicator by the ECEIM 2016 consensus on inflammatory airway disease [1].

An alternative staging system for ambulatory practice that included lung function assessment was later developed [18]. This method included data of the horses’ physical examination (clinical score), BALF cytology (neutrophil percentage), arterial blood oxygen pressure (PaO_2_), pleural pressure (ΔPpl) and histamine bronchoprovocation (maximum tolerated concentration). All the diagnostic procedures could easily be performed in the field, since all the equipment used was portable. However, this method has yet to be validated and the Open Pleth™ used for lung function assessment during the histamine bronchoprovocation is no longer commercially available [2,18].

Staging methods are important tools for monitoring disease progression and treatment response [94]. In equine medicine, asthma staging still relies on invasive ancillary tests which may limit their routine application. For this reason, disease monitoring tends to rely on clinical scores or on owner reported information. We expect that with technological progress, less invasive and more sensitive ancillary tests will become available which will help the equine practitioner make informed decisions in their everyday practice.

### 2.7. SEA Characterization

Although the role of immunoglobulin E (IgE) in the pathophysiology of SEA remains controversial, differences in allergen-specific IgE concentrations in the sera and BALF between healthy and SEA-affected horses have been reported [26,140,141,142,143]. The measurement of allergen-specific IgE concentrations has helped ascertain the association between SEA and sensitization to fungi and mites [26,140,144]. Furthermore, using a microarray platform, White and colleagues have reported that severely asthmatic horses can present different allergen sensitization profiles which usually involve exposure to fungi, mite, pollen proteins and, surprisingly, latex [20].

Intradermal tests (IDT) are used to assess the patient’s reaction to an allergen. It requires the injection of specific allergens intradermally and if the horse is sensitized, a local allergic reaction occurs (papule). In SEA-affected horses the use of IDT allowed the identification of allergen sensitization [43,141]. Lo Feudo and colleagues reported that insects, the mite *Dermatophagoides* spp. and dog epithelium were the major allergen profiles associated with SEA [43].

In human medicine, skin prick tests (SPT) are commonly used in the diagnosis of allergic diseases since they are less invasive than IDT and also provide information about allergen-specific sensitization profiles. These ancillary tests can easily be performed in the horses in a clipped area of the neck by placing a drop of a specific allergen extract and penetrating the epidermis with a lancet. SPT successfully identified allergen sensitization in severely asthmatic horses [42], and has been proposed as a useful diagnostic tool for horses with insect bite hypersensitivity [145].

However, the IDT and SPT are not fully standardized in equine medicine. In IDT, a subjective method based on the wheals’ size, discomfort, thickness, erythema and turgidity has been used to assess the grade of the reaction [43,146]. Nevertheless, in SPT, two very similar cut off values have been found for their use in horses, 1 cm [42] and 0.9 cm [145], which may already guide clinicians in the interpretation of these tests. SPT papule diameter values are calculated as the average of two orthogonal diameters. 

Nonetheless, the measurement of allergen-specific IgE concentrations along with intradermal testing (IDT) and skin prick testing (SPT) allows the recognition of allergen-specific sensitization profiles which can be used to assist in the development of individualized allergen-avoidance protocols.

## 3. Diagnostic Flow-Chart

**Figure 1:**
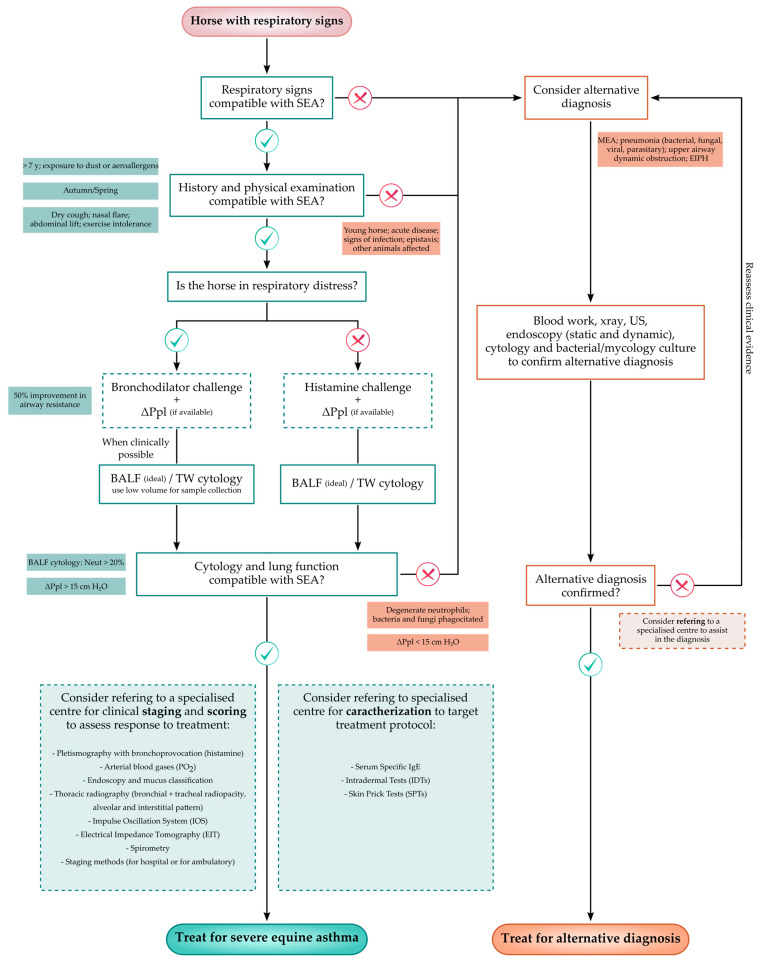
Flow-chart proposal for the diagnosis of severe equine asthma to be used in everyday veterinary practice [17,18,42,43,55,71,117]. MEA—Mild/Moderate Equine Asthma; EIPH—Exercise Induced Pulmonary Hemorrhage; BALF—Bronchoalveolar Lavage Fluid; TW—Tracheal Wash; ΔPpl—Change in pleural Pressure (indirect); SEA—Severe Equine Asthma; and Neut—Neutrophils.

Because horses affected by severe equine asthma may not exhibit clinical signs during disease remission, disease diagnosis can be challenging, particularly to the less experienced practitioner. Thus, a flow-chart which aims to assist in the recognition of severely asthmatic horses by including the main steps required to correctly diagnose the disease, is proposed in Figure 1.

Although SEA is a common respiratory disease in equids, the clinician must be aware that other respiratory conditions, such as mild/moderate equine asthma, may result in lower airway inflammation which might mimic SEA in remission [1,147]. Furthermore, to a less trained eye, a bacterial/fungal pneumonia can be misdiagnosed as SEA, resulting in dire consequences should the affected horse be treated with corticosteroids [148]. A careful physical examination and owner interview, paired with environmental inspection are essential to prevent misdiagnosis. In SEA, the horses’ respiratory signs will be recurrent, and in some cases irreversible, with owners reporting that they might worsen in specific seasons or when the animal is exposed to certain stimuli. Dry cough, respiratory effort with an abdominal pattern, exercise intolerance and nasal discharge are usually reported by owners [1,17,39,46,49]. Because these clinical signs are not pathognomonic of SEA, a thorough physical examination will help rule out other possible causes. The HOARSI questionnaire can be used to collect relevant information on the clinical history of the patient and help identify trigger factors [39]. Similarly, other clinical scores published by different research groups might be helpful in tracking the severity of clinical signs [53,65,66,67,68,69].

Severely asthmatic horses will invariably exhibit airway inflammation, hyperreactivity and obstruction, and disease severity will determine the magnitude of these reversible findings [1]. When in remission, severely asthmatic horses will maintain low-grade inflammation and hyperreactivity but have little or no airway obstruction [18,46,104]. As previously mentioned, lung function tests are the best ancillary diagnostic tools for recognizing these cases. Lung function testing is not yet widely available in equine ambulatory practice and the most sensitive tests for detecting low-grade inflammation can only be found in research centers. However, clinicians can easily measure ΔPpl, using an esophageal balloon catheter and a pressure transducer, in order to assess the degree of airway obstruction [53,93]. The authors use Ventiplot™, a portable pleural pressure measurement device developed by Johannes Peter Schramel, Vetmeduni, Vienna. In order to improve the sensitivity of this test, histamine bronchoprovocation is used which allows the identification of horses in remission, since these animals will maintain some degree of airway hyperreactivity. This procedure should not be performed in horses with respiratory distress, since it will further impair the animal’s ventilation. Alternatively, ΔPpl can be coupled with a bronchodilator challenge and the clinicians should expect a 50% improvement of lung function readings [93].

If possible, cytology should always be performed on SEA-suspected horses. Lung neutrophilia is the hallmark of SEA, and BALF cytology will reveal a differential count of >20% neutrophils [1,149]. Cytology can also help rule out other causes of lower airway inflammation, such as mild/moderate equine asthma (>5% neutrophils and presence of mast cells) or infection (presence of degenerated neutrophils, bacteria and/or fungi) [1,150,151]. It is recommended to always perform cytology in combination with lung function testing since it has been reported that some severely asthmatic horses might have a paucigranulocytic cytological profile [83]. Additionally, when possible BALF cytology should be performed instead of TW to obtain a clearer image of the immune cells found in the horse’s lungs [123,136]. Performing an endoscopically guided BALF collection allows the use of a small volume of fluid for sample collection, which can be useful in patients where instilling a large volume of saline is not recommended. The clinician should also be aware that when tracheal wash is used, the cut off of 20% neutrophils indicates only the presence of airway inflammation [128].

Thoracic radiography, ultrasonography and respiratory endoscopy can also contribute to disease diagnosis, not only by helping exclude alternative diagnostic possibilities but also by enabling SEA staging (endoscopic and radiographic examination) [17,18,46,81]. Whenever possible, these ancillary tests should be performed, although they are not an essential part of the basic diagnostic panel for this disease. Endoscopy can be used to evaluate tracheobronchial mucus accumulation, which in extreme cases will further compromise the animal’s ventilation and might, therefore, constitute a potential therapeutic target for the improvement of clinical signs [54,55,91]. Similarly, performing a thoracic radiography will permit the assessment of the bronchial remodeling in response to bronchoconstriction and lung inflammation. Furthermore, thoracic radiography, ultrasonography and respiratory endoscopy can be performed in the field and this information can be included to help monitor treatment response [71]. Although the findings observed during the thoracic ultrasound of a SEA-affected horse are not specific to this disease, they may be used to assess the severity and chronicity of the inflammatory process. Ultrasound can also assist the clinician in ruling out potential infectious conditions, such as pneumonia/pleuropneumonia.

Although not mandatory, the authors recommend the use of a staging/scoring method to monitor disease progress. SEA-affected animals will require an individualized treatment protocol catered to both horse’s and owner’s needs [29]. Staging can also help the clinician identify potential lack of adhesion to the proposed treatment and can be used as a tool to confront non-adherent owners with the clinical evolution of their asthmatic horse.

## 4. Conclusions

The condition known today as severe equine asthma was first recognized several centuries ago. Since its first description, ancillary diagnostic tests have greatly evolved, but we have yet to develop a single diagnostic tool for disease identification and monitoring. A combination of clinical history, physical examination, lung function assessment and lower airway cytology are therefore fundamental to correctly diagnose affected animals.

Decision making, such as selecting the most suitable diagnostic approach, is a fundamental part of evidence-based medicine and can therefore make the difference between success and failure when managing a clinical case. We propose the use of a simple flow-chart diagram to help clinicians obtain the minimum database required for the diagnosis of SEA in their routine practice. Nonetheless, when possible, other ancillary tests should be performed to stage and characterize each clinical case, in order to improve diagnostic and treatment accuracy. These tests can also assist in monitoring the occurrence of pathological changes, such as mucus accumulation and bronchial smooth muscle hypertrophy secondary to the disease.

## Data Availability

Not applicable.
